# Myocardial Infarction Thought to be Provoked by Local Epinephrine Injection During Endoscopic Submucosal Dissection

**DOI:** 10.4021/jocmr565w

**Published:** 2011-05-19

**Authors:** Hyung Hun Kim, Moo In Park, Seun Ja Park, Won Moon

**Affiliations:** a Department of Internal Medicine, Kosin University College of Medicine, Busan, Korea

## Abstract

Due to its hemostatic effect, local epinephrine has been used to minimize mucosal bleeding during endoscopic submucosal dissection (ESD), but its clinical benefit remains unclear. On the other hand, several adverse events deemed to be caused by epinephrine have been observed during ESD. A 73-year-old woman developed myocardial infarction after ESD for a large rectal adenoma, and an 80-year-old woman developed abrupt chest tightness during ESD. In both patients, changes on electrocardiogram and elevated cardiac markers provided evidence of myocardial ischemia. The patients were transferred to the cardiac care unit and recovered completely. Up to our knowledge this is the first report of myocardial infarction thought to be provoked by submucosal epinephrine injection during ESD.

**Keywords:**

Adenoma; Colon; Endoscopic surgical procedure; Epinephrine; Myocardial infarction

## Introduction

Endoscopic submucosal dissection (ESD) is a minimally invasive technique that is safe, relatively simple, and effective in the curative treatment of early gastric cancer with an extremely low risk of lymph-node metastasis and for the removal of adenomas as precursors to cancer in the gastrointestinal region. Immediate and delayed bleeding are the most frequent complications associated with endoscopic submucosal dissections. Bleeding after gastric ESD is reported to occur in up to 7% of cases [1]. Due to its hemostatic effect, local epinephrine has been used to minimize mucosal bleeding, but its clinical benefit remains unclear. On the other hand, we have observed the following events after epinephrine injection during ESD at our center: high blood pressure, dizziness, headache, palpitations, and even myocardial infarction. Among them, myocardial infarction was the most serious event. Here, we present two cases of myocardial infarction thought to be provoked by local epinephrine injection during ESD.

## Case Reports

### Case 1

**Figure 1.**
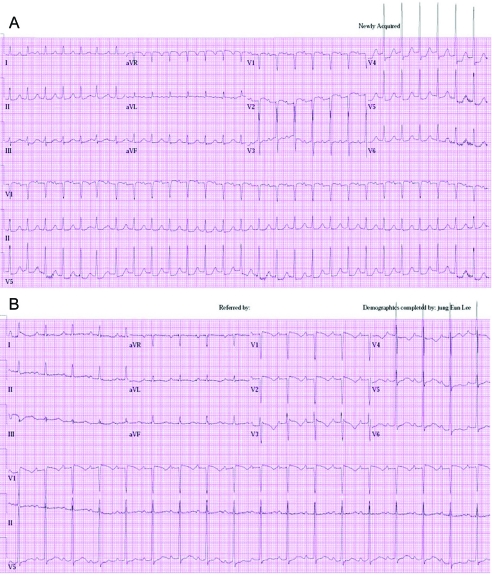
(A) The electrocardiogram showed depressed ST segments from V4 to V6 during the chest pain. (B) The electrocardiogram showed recovery of depressed ST segments from V4 to V6 after resolution of chest pain.

**Figure 2.**
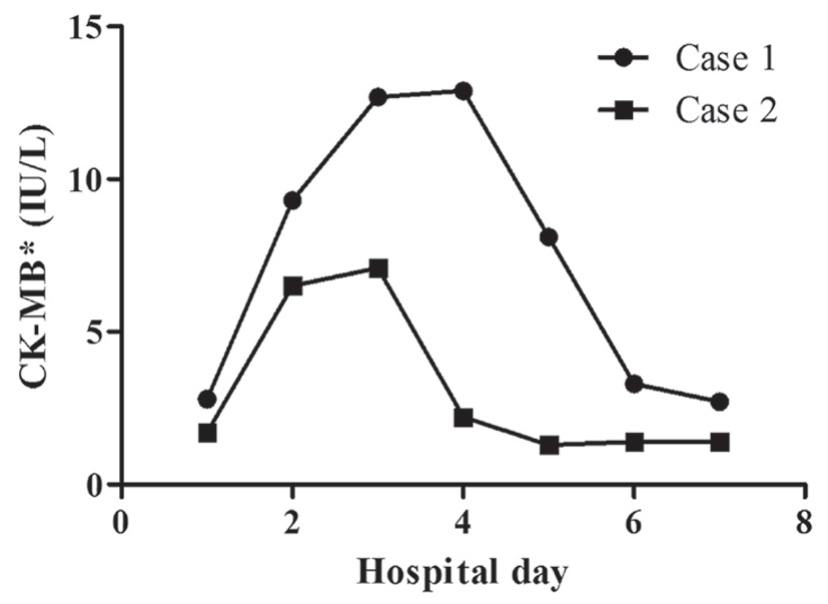
The serial changes of creatine kinase-MB fraction in two cases. * CK-MB: creatine kinase-MB fraction.

**Figure 3.**
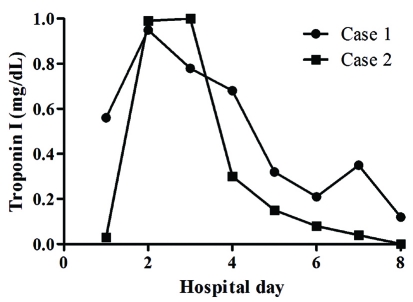
The serial changes of cardiac troponin I in two cases.

A 73-year-old woman was admitted to undergo ESD for a large rectal adenoma detected during routine colonoscopy. It was a laterally spreading tumor, approximately 3.4 cm x 2.7 cm. She had no past medical history. On admission, her vital signs were stable; blood pressure was 110/80 mmHg, heart rate was 70 beats per minute, respiratory rate was 14 breaths per minute, and body temperature was 36.8 ^o^C. The initial electrocardiograph (ECG) and chest radiograph were unremarkable. The usual ESD techniques were used [2, 3]. The patient provided written informed consent before treatment. The patient fasted on the morning of the operation. ESD was performed under conscious sedation. Marks were made 5 mm outside the tumor edge with an argon plasma coagulator (PSD-60, Olympus, Tokyo, Japan), saline-epinephrine (1 : 100,000 solution in saline) was injected into the submucosal layer around the lesion, and the mucosa was cut 5 mm outside the marks. After incision of the mucosa with a Flex knife (KD-630L; Olympus, Tokyo, Japan), submucosal dissection by an Insulation tipped diathermic knife (KD-610L; Olympus, Tokyo, Japan) was performed. Repeated 10 time saline-epinephrine injections were performed to maintain appropriate tissue separation during procedure. All visible vessels on the ulcer floor were coagulated with hemostatic forceps (HDB2422 W; Pentax, Tokyo, Japan). Just after ESD, however, she experienced severe chest tightness and excessive sweating. Her blood pressure was 180/110 mmHg, heart rate was 150 beats per minute, respiratory rate was 22 breaths per minute, and body temperature was 36.8 ^o^C. The ECG showed depressed ST segments from V4 to V6 (Fig. 1A). Decreased lateral wall movement was demonstrated in echocardiograpy, and cardiac makers were significantly elevated; creatine kinase-MB fraction (CK-MB) and cardiac troponin I (cTnI) were 12.90 IU/L and 0.95 ng/mL respectively (Fig. 2, 3). She was transferred to the cardiac care unit with emergent heparinization. Thirty-six hours later, her chest discomfort subsided, with normalization of ECG (Fig. 1B).

### Case 2

**Figure 4.**
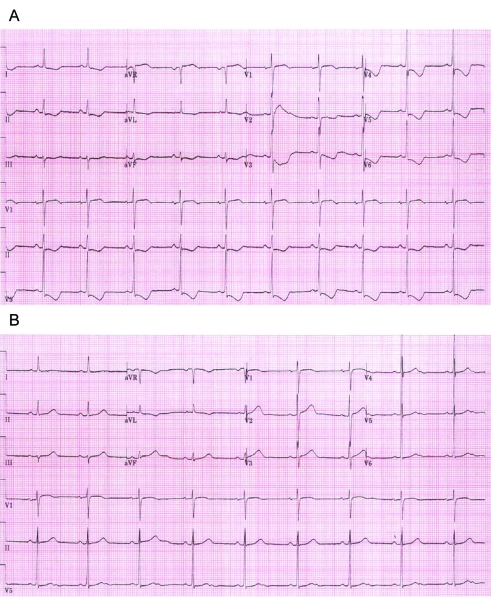
(A) The electrocardiogram demonstrated depressed ST segments at lead II, II, aVF, and from V3 to V6. (B) The electrocardiogram showed normalization of ST segments after recovery from chest pain.

An 80-year-old woman was hospitalized for the removal of a 2 cm x 5 cm rectal adenoma. She had undergone low anterior resection due to left colon cancer, but had no medical history of vascular disease. The initial ECG and chest radiograph showed no remarkable abnormalities, and her vital signs were stable; blood pressure was 100/70 mmHg, heart rate was 60 beats per minute, respiratory rate was 12 breaths per minute, and body temperature was 37.0 ^o^C. ESD was performed as previously described. Just after starting the incision with a Dual knife (KD-650Q, Olympus, Tokyo, Japan) following 60 ml normal saline-epinephrine injection, she developed abrupt left chest discomfort. Her blood pressure was 140/100 mmHg, and pulse rate was 80 beats per min. ECG revealed depressed ST segments at lead II, II, aVF, and from V3 to V6 (Fig. 4A). Echocardiography revealed decreased movement of the lateral wall. Moreover, elevated CK-MB (7.10 IU/L) and cTnI (1.00 ng/mL) were observed (Fig. 2, 3). She was transferred to the intensive care unit, and her symptoms resolved 2 days later with normalization of the ECG (Fig. 4B).

## Discussion

Myocardial infarction during ESD was thought to be caused by three factors. First, ESD requires a large amount of fluid, normal saline solution mixed with epinephrine, to lift lesions from the muscular layer. Second, an abundant vascular network is present in the submucosal layer, so injected epinephrine can be easily absorbed into the systemic circulation. The hemodynamic response to epinephrine infusion has been reported to resemble the cardiovascular response to mental stress [4]. Finally, ESD itself can be a stressful situation for a patient that escalates sympathetic tone. Our patients did not have any known cardiac or other vascular disease. Furthermore, detailed history and physical examination showed nothing remarkable before ESD. However, they developed myocardial infarction evidenced by changes on ECG, decreased wall movement in echocardiography, and increased cardiac makers.

Well-known risk factors for myocardial infarction include abnormal lipids, smoking, abdominal obesity, hypertension, diabetes mellitus, low physical activity, old age, and psychosocial stress factors [5]. Myocardial infarction is the primary cause of death in men older than 45 years of age and in women older than 65 years of age [6]. Both of our patients were over age 65; the patients were 73 and 80 years old, respectively. A large amount of submucosal epinephrine injection might be a factor that can provoke an effect resembling mental stress, a risk factor of myocardial infarction in our old patients [4].

In the case of colorectal polypectomy, two prospective, randomized comparative studies of submucosal injections with and without epinephrine reported that epinephrine in the submucosal injection fluid did not reduce the overall risk of delayed bleeding [7, 8]. In one of the studies, immediate bleeding did occur less frequently in the epinephrine group than in the control group without epinephrine [8]. However, immediate bleeding is generally not as serious as delayed bleeding because it can usually be successfully controlled using the endo-clip technique or electric coagulation methods, such as hot biopsy or argon plasma coagulation. These reports support the opinion of the American Society for Gastrointestinal Endoscopy editorial that stated there is no mandate to include epinephrine in the injection fluid because the overall risk of immediate bleeding is low, and the immediate bleeding can generally be treated successfully by experienced endoscopists.

Adults over age 65 are more likely to die of a myocardial infarction [9]. Moreover, older women are twice as likely to die within a few weeks of a myocardial infarction as men [10]. Considering all these points, we cautiously suggest it would be reasonable to consider injecting only normal saline, not a normal saline solution mixed with epinephrine, to lift lesions for ESD in patients with risk factors for myocardial infarction, especially when they are over 65 years old.
